# Novel Detection of Pleomorphic Adenomas via Analysis of ^68^Ga-DOTATOC PET/CT Imaging

**DOI:** 10.3390/cancers16152624

**Published:** 2024-07-23

**Authors:** Felix Johnson, Marcel Kloppenburg, Benedikt Hofauer, Barbara Wollenberg, Cosima C. Hoch, Fabian Stögbauer, Bernhard Haller, Andreas Knopf, Ulrich Strassen, Susan Notohamiprodjo

**Affiliations:** 1 Department of Otorhinolaryngology, Medical University of Innsbruck, Anichstraße 35, A-6020 Innsbruck, Austria; 2 Department of Otorhinolaryngology, Klinikum rechts der Isar, TUM School of Medicine and Health, Ismaninger Str. 22, 81675 Munich, Germany; 3 Institute of General and Surgical Pathology, Klinikum rechts der Isar, TUM School of Medicine and Health, 81675 Munich, Germany; 4 Institut für KI und Informatik in der Medizin, Klinikum rechts der Isar, TUM School of Medicine and Health, 81675 Munich, Germany; 5 Department of Otorhinolaryngology, Head and Neck Surgery, Albert-Ludwigs-Universität Freiburg, 79106 Freiburg, Germany; 6 Department of Nuclear Medicine, Klinikum rechts der Isar, TUM School of Medicine and Health, 81675 Munich, Germany

**Keywords:** pleomorphic adenoma, carcinoma ex pleomorphic adenoma, Warthin tumor, salivary gland tumors, PET/CT, PET/MRI, DOTATOC, DOTATATE, ^68^Ga, gallium, immunohistochemistry, SSTR2, peptide receptor radionuclide therapy, somatostatin analogue, radiomics

## Abstract

**Simple Summary:**

We present in this study novel data which demonstrates that the most common type of tumor of the salivary glands may be accurately diagnosed using a specific type of radiological imaging. This may be used to help discriminate this tumor or recurrent versions of it amid various types of benign and malignant tumors. Furthermore, this data suggest that new avenues of minimally invasive therapy may be viable for these tumors and potentially even malignant versions of these tumors and should be examined in further studies.

**Abstract:**

Introduction: Currently, the diagnosis of salivary gland tumors using imaging techniques is unreliable. Methods: In this monocentric retrospective study, we examined patients who received a ^68^Ga-DOTATOC PET/CT and subsequently underwent a salivary gland tumor resection between 1 January 2010 and 31 December 2021. PET/CT image assessment was compared with somatostatin receptor (SSTR) expression and histology. Results: Thirteen patients (five pleomorphic adenoma (PA) and eight other parotid lesions (OPL)) received a ^68^Ga-DOTATOC PET/CT. Imaging displayed strong focal tracer uptake in all PA except for one with strong tumor to background discrimination. PA revealed higher SUVmax, SUVmean, liver and blood pool quotients than those of Warthin tumors (WT) and of OPL. In comparison to the contralateral parotid, SUVmax (*p* = 0.02), SUVmean (*p* = 0.02), liver quotient (*p* = 0.03) and blood pool quotient (*p* = 0.03) were all significantly higher. In contrast, WT and OPL showed in relation to the contralateral parotid no significant differences of SUVmax (WT *p* = 0.79; OPL *p* = 0.11), SUVmean (WT *p* = 1.0; OPL *p* = 0.08), liver quotient (WT *p* = 0.5; OPL *p* = 0.08) and blood pool quotient (WT *p* = 0.8; OPL *p* = 0.19). Two PA and one granuloma were not available for examination. In the immunohistochemal analysis, all PA demonstrated the highest intensity of SSTR2 expression (grade 3). Furthermore, PA had a high percentage of cells expressing SSTR2 (20%, 80% and 55%). Conclusions: A strong tracer uptake in PA was shown in ^68^Ga-DOTATOC PET/CT. This may allow physicians to utilize radioligated somatostatin analogue PET CT/MR imaging to accurately diagnose PA. Additionally, it may be possible in the future to treat the PA with a noninvasive peptide receptor radionuclide therapy or with somatostatin analogues.

## 1. Introduction

The pleomorphic adenoma (PA) is considered to be one of most common tumors of the salivary glands and has had a growing incidence in the past 20 years. Though the PA may develop in any salivary gland tissue, the most common location is the parotid gland (85%) followed by smaller salivary glands (10%) and the submandibular gland. The PA has a predilection for women between the ages of 30 to 60 [1].

In the current World Health Organization (WHO) Classification of Head and Neck Tumors: Salivary Glands (5th edition), malignant and benign epithelial tumors are classified into 21 and 15 tumor types, respectively [2]. Histologically, this tumor is described as having epithelial and myoepithelial elements as well as a myxochondroid stroma and is characterized by a mild to moderate cellular pleomorphism. Its name is derived from the myriad parenchymatous architectural characteristics visible under light microscopy. While this tumor is also described as a ‘benign mixed tumor’, the PA has a risk for malignant transformation to carcinoma ex pleomorphic adenoma (CXPA) ranging between 5% and 11.8% over a period of 10 to 15 years [3,4,5,6]. Furthermore, in extremely rare cases, metastasis of PA to bones, lungs or lymph nodes has been documented [7,8]. A total of only 83 cases of metastatic pleomorphic adenoma (MPA) have been reported [9]. In the 4th edition of the WHO classification for head and neck tumors published in 2017, MPA was categorized as malignant [10]. Since the release of the 5th edition of the WHO classification for head and neck tumors in 2022, MPA has been reclassified as a subtype of PA, effectively downgrading it to a variant of benign PA [11]. Additionally, carcinosarcoma has been maintained as a distinct entity in the 2022 WHO classification [11]. Nonetheless, it is hypothesized that carcinosarcomas also originate from PA, representing a more aggressive variant of CXPA [12]. The PA typically presents as a slowly growing, painless tumor, without symptoms of malignancy including pain or facial paralysis. The risk of malignancy has been shown to positively correlate with certain risk factors including female gender and younger age of first diagnosis, length of time the tumor is in situ and a larger tumor size [13]. It is this risk of malignancy alteration as well as the persistent neoplastic growth which necessitate the recommendation for surgical excision. A conservative ‘watch-and-wait’ strategy ultimately leads to a higher risk for malignancy as well as a subsequently larger tumor, which may also be more difficult to remove it surgically [3].

During surgery, extreme caution must be used to avoid incomplete resection, as this may lead to local recurrence [7,8,14,15]. This risk is highly dependent on the expertise of the surgeon, though it is also partially dependent on the histology. The various subtypes of PA include the classic, myxoid and cellular pleomorphic adenomas, with the myxoid subtype having the thinnest pseudocapsule and the highest likelihood for postoperative recurrence [16]. In order to prevent recurrence, the surgeon tries to remove the lesion with a surrounding sheath of healthy tissue. This is typically achieved via complete or partial removal of the gland or extracapsulare dissection. Typically, the smaller and more superficially situated the tumor, the less extensive the required surgical method [17]. For tumors located in the deep lobe of the parotid gland, a total parotidectomy may be indicated. The available data on recurrence rates across different surgical techniques are very poor [18]. The classic parotidectomy involves the surgical preparation and visualization of the facial nerve. The risk of injury to the facial nerve is highly dependent on the tumor size and localization. A much higher risk of facial nerve lesion is found in patients who are operated on more than once, as postoperative scarring makes additional surgery much more difficult. The prognosis for pleomorphic adenomas is estimated to be approximately 95% cure rate postsurgery [19].

Preoperative examination for the removal of a tumor in the head and neck area is essential. The salivary glands may be quickly and inexpensively examined using sonography. In ultrasound, the pleomorphic adenoma displays smooth margins, often with lobules, and either homogeneous or heterogenic parenchyma. Doppler sonography typically shows little vascularization. Calcifications as well as necrosis tend to appear the larger the tumor becomes [20,21]. The skill of the sonographer is important in interpreting the nature of the tumor. Using ultrasound alone the accuracy of determining a PA is described as 64% and of determining a Warthin tumor as 82% [22]. A large meta-analysis of fine-needle biopsy studies found a sensitivity between 70.4 and 88% and a specificity between 99.5 and 98%. Although sensitivity and specificity of ultra-sound guided FNA are quiet high negative, positive predictive values still remain unsatisfactory (85.3–94.1%) [23]. Therefore, parotid surgery cannot be avoided to assure correct identification of parotid malignancies.

The main limit for ultrasound examination of the salivary glands is when tumors spread into the parapharyngeal space, as the extent of spread may be more difficult to visualize.

**Figure 1:**
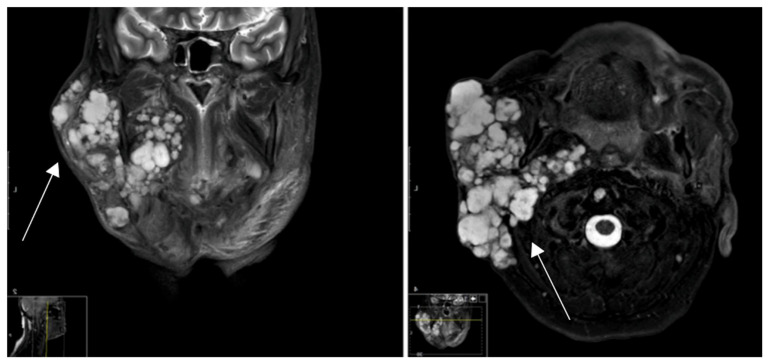
MRI. Multilocular local tumor recurrence and extra parotid tumor spread of a pleomorphic adenoma by status post-R1-resection many years earlier.

Radiologic examination of pleomorphic adenomas is usually performed using CT or MRI (Figure 1) and may give valuable information when tumors are in the deeper parotid lobe or when they develop from the smaller salivary glands. While the PA may display either a heterogenous or homogenous parenchyma they have a positive trend towards developing a more heterogenic parenchyma, including the presence of calcification and necrosis, as tumor volume increases [24,25].

While the resolution of ultrasound, MRI and CT have improved, the ability to accurately differentiate tumors of the salivary glands has not advanced significantly. The goal of accurately identifying and discriminating tumors of the salivary glands has largely been abandoned due to the reasoning that regardless of the entity, the recommendation towards surgical excision will remain unchanged. However, this reasoning is logically faulty, it assumes that accurate diagnostic imaging for a specific tumor such as the PA is not possible and that surgery is the only possible treatment.

However, a case-study described the incidental and previously undescribed strong tracer uptake in a pleomorphic adenoma in a ^68^Ga-DOTATOC PET/CT, and hereby implied the presence of the somatostatin receptor 2 (SSTR2) in the PA [26]. The first steps towards utilizing diagnostic tools in identifying PA include thoroughly understanding their histocytology. A recent study [27] verified the previously undescribed strong presence of the somatostatin receptor 2 in pleomorphic adenomas via immunohistochemistry. An analysis of 306 tumors including 207 nonPA and 99 PA using the HER2-mama scale for the assessment of SSTR2 expression demonstrated that PA strongly express the SSTR2. This research provides the fundamental biomolecular explanation for the tracer uptake in the previously described PA.

Somatostatin and the somatostatin receptor have been shown to play a role in inhibiting angiogenesis and cell growth [25]. Various SSTR subtypes exist and these are expressed in different types of tissue including benign and malignant neoplasms [28] and inflammatory processes [29]. Nuclear radiologic examinations use this feature to specifically ligate radioisotopes to tissues and tumors expressing the somatostatin receptor. The correlation of SSTR2 expression with ^68^Ga-DOTATOC uptake has been confirmed [30]. Neuroendocrine tumors (NET), as well as some autoimmune disorders may be visualized using the PET/CT or PET/MRI. However, further studies investigating this incidental tracer uptake in PA have not been performed. If tracer uptake in PA could be observed in other PA, an avenue to differentiate and possibly treat the PA would open. This study aimed to assess the impact of ^68^Ga-DOTATOC PET/CT to discriminate the SSTR2 expression of pleomorphic adenoma from that of other types of salivary gland tumor.

## 2. Methods

### 2.1. Patient Selection and Data Collection

In this monocentric retrospective study, we included NET patients of the University Hospital rechts der Isar of the Technical University of Munich who underwent ^68^Ga-DOTATOC PET/CT with incidental findings in the parotid gland and subsequently underwent a salivary gland tumor removal operation between from January 2010 and December 2021. Based upon these selective criteria, a total of 13 patients were included in the study. Histology data were extracted from the electronic patient database (SAP) of the University Hospital rechts der Isar. Patient information was de-identified and systematically entered into an Excel (V16.19) spreadsheet. The study protocol was in accordance with the Declaration of Helsinki and received approval from the Institutional ethics board of the Medical Faculty, Technical University of Munich.

### 2.2. Immunohistochemistry Protocol

For immunohistochemistry (IHC) analysis, 3 µm thick sections of formalin-fixed and paraffin-embedded (FFPE) tissue blocks were prepared. These sections were incubated into an SSTR2A antibody (ZYTOMED Systems, Berlin, Germany; 1:100) for 32 min. Staining was performed using a Ventana BenchMark Ultra automated stainer with the iView DAB Kit were utilized for immunohistochemical stainings (Ventana Medical Systems, Oro Valley, AZ, USA). Finally, tissue sections were counterstained with hematoxylin and positive controls were included for quality assurance.

### 2.3. IHC Scoring Methodology

**Figure 2:**
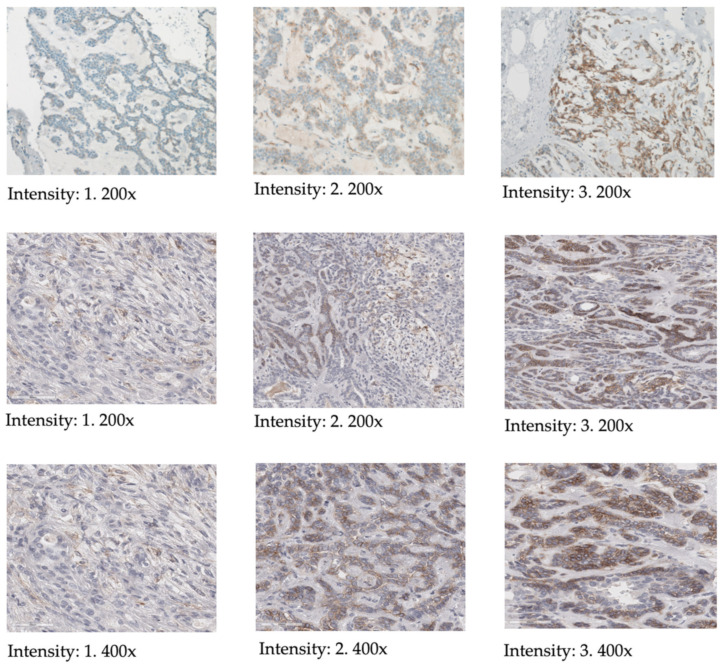
Illustration of SSTR2 staining and scoring of the SSTR2 intensity according to the HER2 scoring system, including magnification [27].

IHC scoring was conducted using light-microscopy, employing the HER2 scoring-system typically used for breast cancer hormone receptor analysis (see Table 1 and Figure 2) [31]. Membranous staining was identified as specific, with evaluation criteria adapted from methods previously described for pancreatic neoplasms [24,32]. Scoring was performed as follows: score 0 for no or weak membranous staining in <10% of cells, score 1+ for weak membranous staining in >10% of cells, score 2+ for moderate membranous staining in >10% of cells and score 3+ for strong membranous staining in >10% of cells. Additionally, the percentage of cells of the tumor expressing SSTR2A (ranging from 0 to 100%) as well as the intensity of staining (0 = none, 1 = mild, 2 = moderate, 3 = strong) were noted for each tumor.

### 2.4. Acquisition, Analysis and Interpretation of Images

All patients received an injection with ^68^Ga-DOTATOC and were examined approximately 15 to 20 min postinjection with a Biograph mCT64 PET/CT machine (Siemens Healthineers, Erlangen, Germany). The CT protocol selection was based on the clinical indication. All examinations included a CT scan from neck to pelvis with the patient’s arms elevated, facilitating anatomical correlation and attenuation correction. Depending on the clinical requirement, the scans were either contrast-enhanced in the portal-venous phase (120 kV, 180 mAs) or conducted using a low-dose protocol without contrast enhancement (120 kV, 25 mAs). Patients with head-neck tumors received a contrast-enhanced CT of the head and neck with arms positioned downwards (120 kV, 180 mAs). Multiplanar reformations were generated with 3 mm slice thickness and 1 mm slice thickness for lung imaging. PET images were obtained using time-of-flight technique with 1.5 mm per second.

All images were interpreted and analyzed by a resident from the department of nuclear medicine. Tracer uptake in PET/CT images was determined using both qualitative and semi-quantitative imaging techniques. Qualitative analysis was achieved by conducting a visual inspection of tracer uptake in reference areas with no pathology and comparing them to uptake in tumors. This process generated “tumor-scores”, which allowed a comparison of nonpathological with pathological tissue and a grading of tracer uptake in terms “lower”, “equal” and “higher” than the reference tissue. Semi-quantitative analysis of images was performed using a standardized uptake value (SUV) algorithm, allowing calculation of a tracer uptake ratio “Lesion SUV/Reference SUV”.

All image analyses were performed using Syngo.via (Siemens Healthineers, Erlangen, Germany). The SUVmax and SUVmean were measured in the parotid tumor, the contralateral healthy parotid parenchyma, liver and mediastinal blood pool using volumes of interest (VOI). Ratios were calculated to compare SUVmax in the tumor (or contralateral parotid) to SUVmean in liver parenchyma and mediastinal blood pool, termed liver quotient and blood pool quotient, respectively. Given the variability in SUV values due to different technical aspects (e.g., imaging equipment, acquisition time postinjection) and patient conditions (e.g., clinical and oncological status), ratios were computed between the SUV values (SUVmax, SUVmean, liver quotient and blood pool quotient) of the tumor and corresponding contralateral reference sites (Ratio-SUVmax, Ratio-SUVmean, Ratio-liver quotient and Ratio-blood pool quotient). This approach aimed to mitigate intrinsic dependencies as previously reported [33,34].

### 2.5. Statistical Analysis

Continuous variables were analyzed via descriptive statistics including arithmetic mean, standard deviation, median and range, whereas categorical variables were investigated via frequencies. To assess the statistical significance of differences between groups, either a paired *t*-test or *t*-test for independent samples with different variances, was employed, as appropriate. The threshold for statistical significance (α) was set to 0.05.

Receiver operating characteristics (ROC) analysis was performed to compare the percentage of cells staining positive for SSTR2 and intensity for SSTR2 and the intensity of SSTR2 uptake between pleomorphic adenoma and other parotid lesions.

## 3. Results

**Figure 3:**
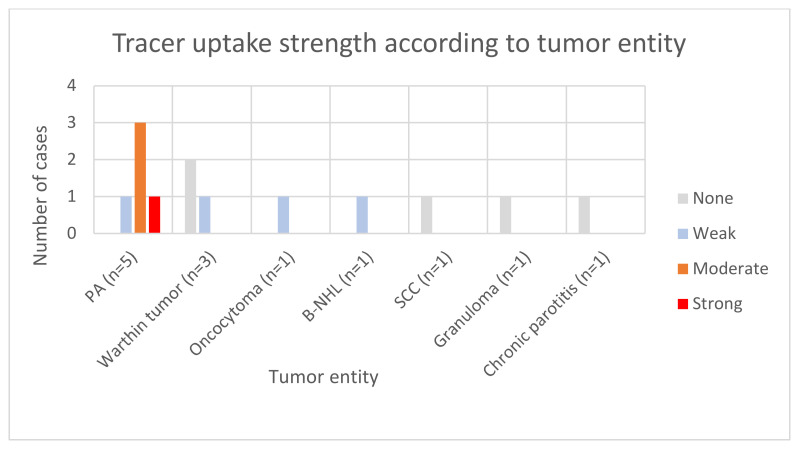
Tracer uptake strength per tumor entity.

A total of thirteen patients received a ^68^Ga-DOTATOC PET/CT scan followed by a salivary gland tumor removal. In five patients the parotid tumor was right-sided and left-sided in eight patients. The histological classification of the tumors included five tumors pleomorphic adenoma, three Warthin tumors and five other parotid lesions, namely oncocytoma, B-cell non-Hodgkin Lymphoma (B-NHL), squamous cell carcinoma, chronic parotitis and granuloma. In the DOTATOC PET/CT imaging, two PAs demonstrated a strong tracer uptake, two others showed moderately strong uptake and one PA demonstrated weak uptake. Additionally, one oncocytoma and one B-NHL case displayed weak tracer uptake (see Table 2 and Figure 3).

The average DLP of the low dose protocol was 148.8 mGy*cm. The average DLP of the standard protocol with additional head and neck CT scan was 1306.7 mGy*cm. An average of 116.58 MBq ^68^Ga-DOTATOC was injected.

**Figure 4:**
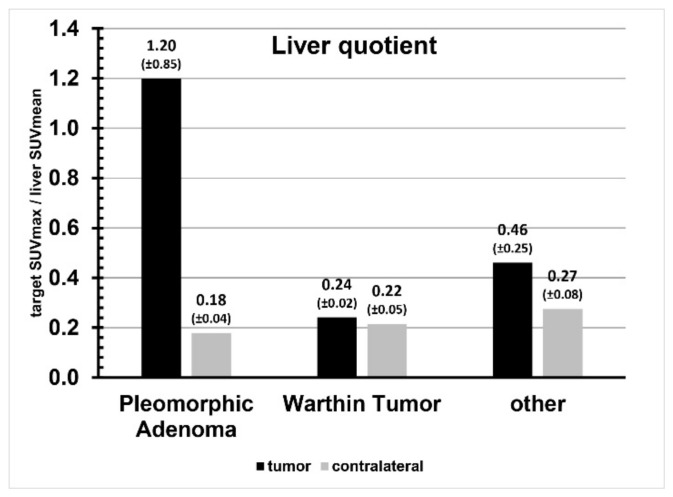
Liver quotient comparison per tumor entity.

**Figure 5:**
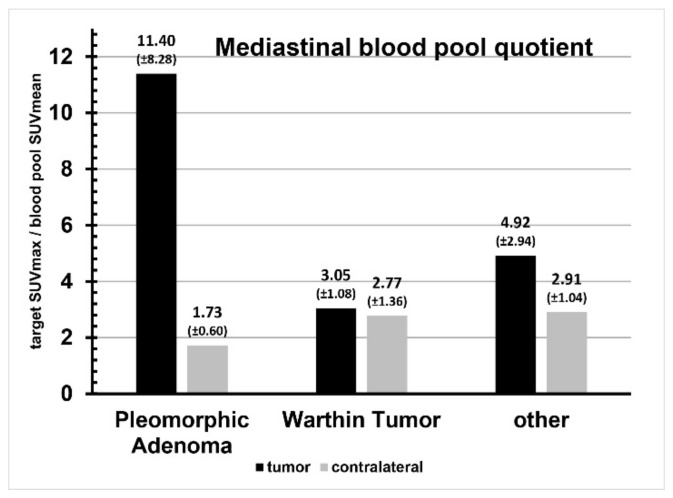
Mediastinal blood pool quotient comparison per tumor entity.

**Figure 6:**
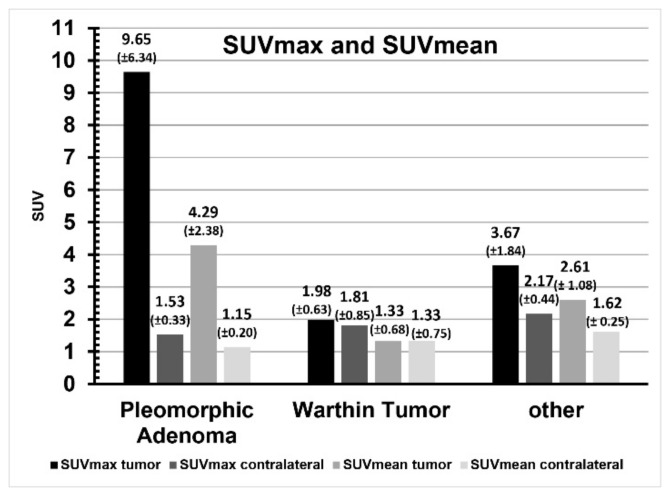
SUVmax/SUVmean comparison per tumor entity.

Enhancement of the liver was SUVmax 10.63 ± 2.13 and SUVmean 8.37 ± 1.46. The tumor-to-liver quotient is shown in Figure 4, the respective enhancement of the mediastinal blood pool was SUVmax 1.52 ± 0.65 and SUVmean 0.81 ± 0.20. The tumor-to-mediastinal blood pool quotient is shown in Figure 5. Enhancement of the healthy contralateral parotid gland was SUVmax 1.84 ± 0.56 and SUVmean 1.37 ± 0.42. The relation of tracer uptake of the tumor and of the contralateral parotid gland tissue is shown in Figure 6. A comparison of SUVmax and SUVmean, as well as liver and blood pool quotients, and the data from the ratio comparisons is visible in Table 3, Table 4 and Table 5.

In pleomorphic adenoma SUVmax, SUVmean, liver quotient and blood pool quotient were significantly higher than in the contralateral parotid gland (all *p* < 0.05). In contrast, Warthin tumor and other parotid lesions showed no significant differences in SUVmax, SUVmean, liver quotient and blood pool quotient when compared to the contralateral parotid.

**Figure 7:**
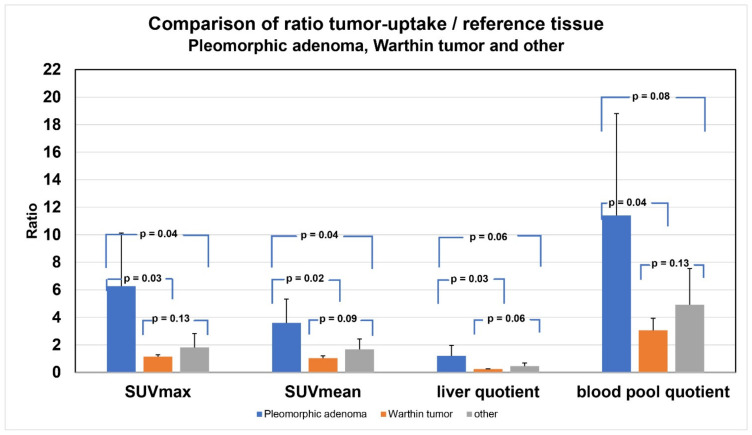
Comparison of the ratio of tumor uptake to reference tissue.

Direct comparison of the ratio tumor uptake/reference tissue between pleomorphic adenoma, Warthin tumor and other parotid lesions revealed significantly high tumor uptake values in pleomorphic adenoma in terms of SUVmax, SUVmean, liver quotient and blood pool quotient, particularly if compared to the Warthin tumor (Figure 7). However, compared to other parotid lesions, tumor uptake values in pleomorphic adenoma were significantly higher in terms of SUVmax and SUVmean, but not significant in terms of liver quotient and blood pool quotient. Tumor uptake values in Warthin tumor were all less compared to other parotid lesions, but with nonsignificant differences in terms of SUXmax, SUVmean, liver quotient and blood pool quotient.

**Figure 8:**
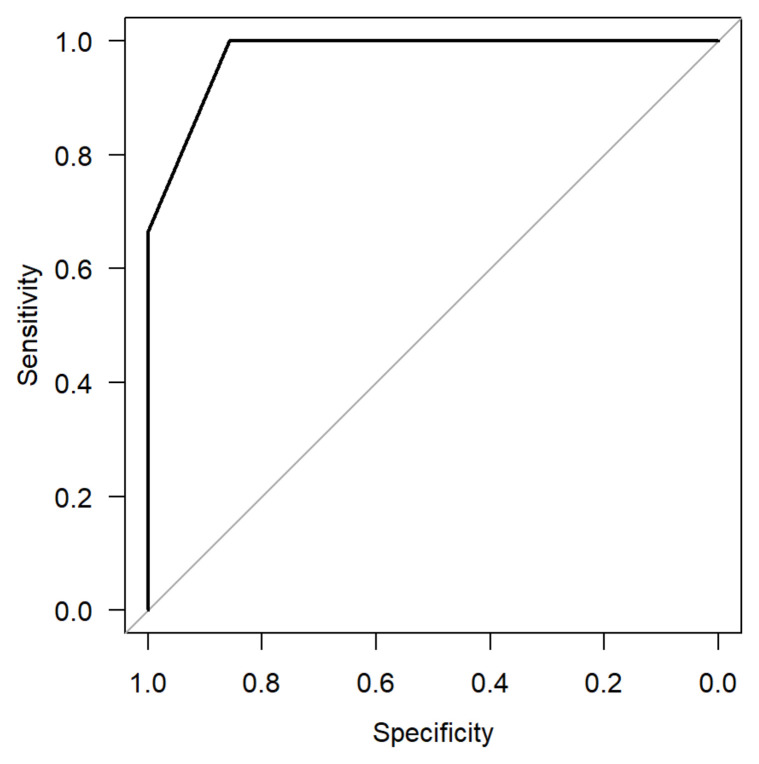
Area under the curve (AUC: 0.9762, 95% CI: 0.86–1) analysis comparing pleomorphic adenoma vs. other parotid lesions for the percentage of cells staining positive for SSTR2.

**Figure 9:**
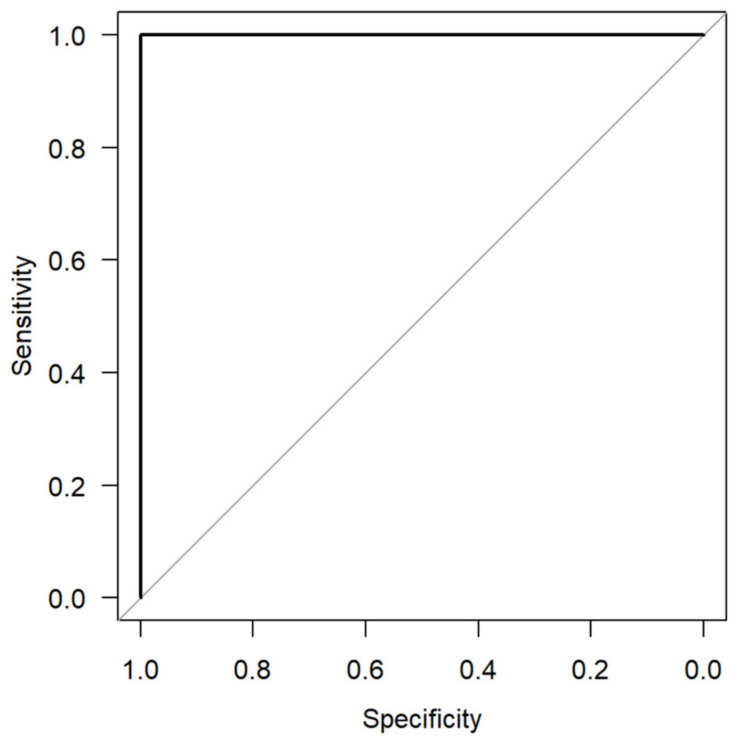
Area under the curve (AUC: 1.0) analysis comparing pleomorphic adenoma vs. other parotid lesions for the intensity of SSTR2 staining (using HER2 mama scale).

Figure 8 and Figure 9 present the AUC to compare PA to other parotid lesions concerning the number of cells staining positive for SSTR2 and intensity of SSTR2 staining. The AUC for the number of total cells staining for SSTR2 was found to be 0.98 and 0.93 for the intensity of SSTR2 staining.

**Figure 10:**
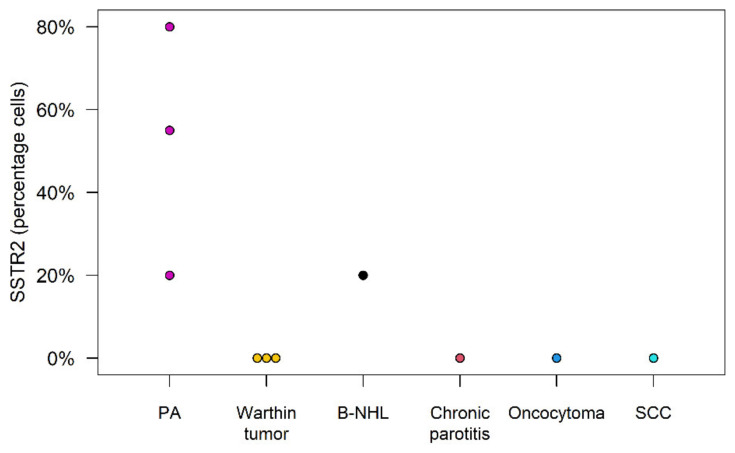
Percentage of cells staining positive for the SSTR2 in IHC.

**Figure 11:**
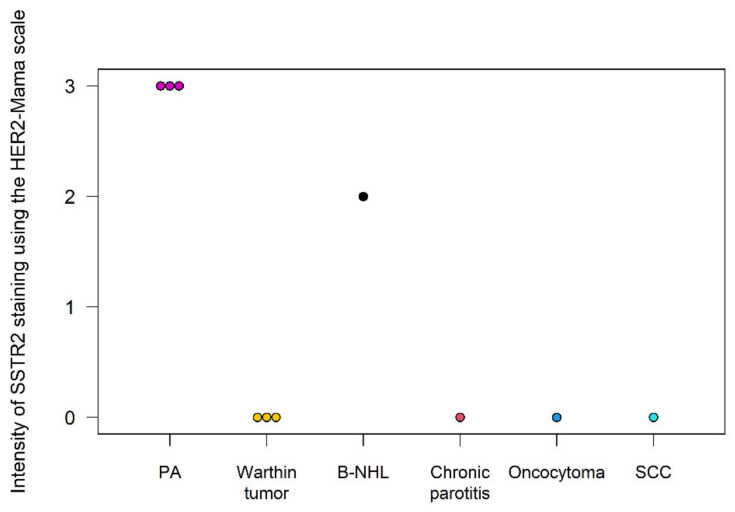
Intensity of SSTR2 staining in IHC.

Immunohistochemistry analysis demonstrated a strong presence and intensity of the SSTR2 in PAs. Of the nonPA tumors, only the B-NHL exhibited a 20% cell positivity for SSTR2 and an intensity of 2. All other parotid lesions showed no cells staining for the SSTR2 (see Figure 10 and Figure 11).

## 4. Discussion

In our study, the SUVmax, SUVmean, liver quotient and blood pool quotient in pleomorphic adenomas was found to be significantly higher than those in Warthin tumor (WT) and other parotid lesions as well as in relation to the contralateral parotid gland (all *p* < 0.05). In contrast, in relation to the contralateral parotid gland, the differences of SUVmax, SUVmean, liver quotient and blood pool quotient in WT and other parotid lesions were not significant. This translates to a significantly stronger tracer uptake and thus suggests enhanced expression of SSTR2 in pleomorphic adenomas as compared to the other examined lesions. To create a scale of benign and malignant parotid tumors derived from ^68^Ga DOTATOC uptake further studies are needed.

In IHC analysis, the B-cell non-Hodgkin lymphoma was the only tumor to demonstrate a positivity for SSTR2 presence (20%) with an intensity of SSTR2 (grade of 2), which explains its weak tracer uptake in DOTATOC PET/CT. The oncocytoma demonstrated no SSTR2 expression and yet it showed a weak tracer uptake. In contrast, all pleomorphic adenomas had a strong SSTR2 staining intensity (grade 3) and showed a high proportion of positive stained SSTR2 cells (20%, 55%, 80%) in comparison to other parotid lesions.

Salivary glands may develop a wide range of different neoplasms. While sonography, MRI and CT are generally effective in differentiating between benign and malign tumors, a distinguishing among benign tumors remains a challenge. A case study described strong tracer uptake in a PA which was incidentally examined during a DOTATOC PET/CT [26]. This tracer uptake suggests that PA strongly express the SSTR2. This was subsequently verified by our group, which examined the presence of SSTR2 in pleomorphic adenoma [27].

The SSTR2 is expressed to varying degrees across different tissues. Some tumors, such as neuroendocrine tumors, express the SSTR2 strongly, which allows a sensitive detection of said tumors and their metastases. Aside from its diagnostic value, a high expression of the SSTR2 opens therapeutic avenues, such as the noninvasive nuclear peptide therapy. The advent of somatostatin analogues (SSA), which do not degrade as quickly as the native somatostatin peptide, provided physicians a method for treating SSTR2 expressing tumors. These SSAs may be ligated to radiopeptides, which may then together lead to a targeted local radiotherapy via alpha and beta particles of SSTR2 expressing tumors [35]. The most typical SSAs include “DOTA-peptides” such as DOTATOC (DOTA-Tyr3-octreotide) and DOTATATE (DOTA-Tyr3-octreotate). The ligation to radiopeptides including gallium-68 (^68^Ga), which has a short half-life, leads to the radioactive decay and the emission of positrons. The detection of positron emission from tumors expressing SSTR2 using positron emission tomography allows the specific visualization of tumors.

The radioactive exposure which results due to this imaging is not significantly more than a typical CT. The actual effective radiation dose for the ^68^Ga-somatostatin PET/CT study averages less than 5 mSv, less than the 7.7 mSv from a CT of the abdomen [36]. Further research and advents in radiopharmaceuticals including ^64^Cu-DOTATOC demonstrate progress in improving spatial resolution with stronger tumor-background contrast [37]. The field of PET imaging and therapy using pharmaceutical radioisotopes is expected to advance, potentially reducing costs and increasing utility.

Originally, the diagnostic test of ^68^Ga DOTATOC was carried out for patients with other malignant tumors, especially NETs and the authors have seen a high tracer uptake in PAs. The approval for clinical use of ^68^Ga DOTATOC in Germany is for staging of NET. In our study, we examined patients with this tracer who subsequently underwent a salivary gland tumor resection because of suspicion of malignancy. The application of ^68^Ga DOTATOC PET/CT for indications other than NET is “off-label use” or for research purposes and require official governmental permission. ^68^Ga-somatostatin PET/CT costs vary depending on the clinic and state, but the cost in our clinic is approximately EUR 2000 in the setting of the German health care system. Although costly, DOTATOC-PET/CT might allow the preoperative precise diagnosis of CXPA which in turn might allow a minimally invasive targeted therapy in the future. This would prevent unnecessary parotid surgery and the associated surgical complications such as facial nerve palsy, Frey syndrome and salivary fistula.

Our IHC analysis corroborates that PAs strongly express SSTR2. This study is the first to demonstrate the potential of DOTATOC or DOTATATE PET/CT imaging in accurately differentiating PAs, one of the most prevalent tumors of the salivary glands. Theranostics is an elegant noninvasive approach of using one radioactive drug to identify and a second radioactive drug to deliver a therapy to a tumor or its metastases. This targeted radiotherapy has been long applied in treating tumors in anatomically sensitive locations such as the thyroid and pituitary glands, as well as metastatic neuroendocrine tumors. Utilizing the strong SSTR2 expression in PAs, theranostics could provide diagnostic clarity and an alternative to surgery via noninvasive peptide receptor radionuclide therapy (PRRT). An accurate diagnosis may allow surgeons to advise patients concerning an alternative therapy to an operation. Instead of having a risky re-operation associated with an increased risk of facial nerve injury due to scarring [37,38], a targeted radiotherapy may be performed. A further indication for a minimal invasive theranostic treatment may be patients with multiple comorbidities and an increased risk for anesthesia. Furthermore, malignant tumors like the carcinoma ex pleomorphic adenoma (CXPA) with metastases may be given a target radiotherapy, or as a neoadjuvant treatment for large, difficult to operate tumors. The treatment of neuroendocrine gut tumors using ^177^Lu-DOTATATE has been shown to significantly improve overall progression free survival [39]. Such a treatment may be applied to patients with CXPA with metastases to be investigated in further studies.

Somatostatin is a neuropeptide which has multiple functions including inhibiting growth hormone. In its native state it is quickly degraded and only has a short half-life of under 3 min. Synthetically produced somatostatin analogues (SSA) such as octreotide and lantreotide have a much longer half-life and may be used to inhibit the growth of tumors expressing the SSTR2 [40]. Prospective placebo-controlled and randomized studies have shown that neuroendocrine tumors displaying a stronger SSTR2 expression are more likely to have a favorable prognosis when treated using SSAs [41,42,43]. Such a trend may also be seen in the CXPA, which have both been shown to have a high percentage of cells expressing SSTR2 as well as a strong intensity of SSTR2 staining. This therapy is introduced when patients have a morbid prognosis including cases of metastasis. The discovery of the SSTR2 in PA and CXPA could allow patients with multiple comorbidities, a morbid prognosis, or nonoperable CXPA tumors to receive a conservative treatment.

## 5. Conclusions

We were able to confirm via IHC the presence and strong expression of the somatostatin receptor (subtype 2) in pleomorphic adenomas. The percentage of cells expressing and intensity of staining of SSTR2 receptor in PAs was summarily higher than in all other parotid lesions. The PAs examined in this study exhibited a stronger tracer uptake as compared to other parotid lesions. In pleomorphic adenoma the SUVmax (*p* = 0.02), SUVmean (*p* = 0.02), liver quotient (*p* = 0.03) and blood pool quotient (*p* = 0.03) were all significantly higher than in the contralateral parotid gland, whereas this was not the case for other parotid lesions including the Warthin tumor. This study confirms the strong tracer uptake in PA in DOTATOC PET/CT imaging. This attribute might be useful in the targeted nonsurgical treatment of carcinoma ex pleomorphic adenoma in the future. Further prospective studies should be performed to corroborate these findings.

## Figures and Tables

**Table 1:** IHC Analysis using a HER2 scoring system, modified according to Tripathy, Mishra et al. 2018 [31].

Staining	Score	Evaluation
No staining observed, faint membrane staining in ≤10% of tumor cells	0	None
Incomplete, barely visible staining in >10% of tumor cells	1	Mild
Incomplete and/or weak circumferential staining in >10% of tumor cells, or complete, intense staining in ≤10% of tumor cells	2	Moderate
Complete, intense, staining in >10% of tumor cells	3	Strong

**Table 2:** Summary of patient collective data.

Patient	Entity	Percentage of Cells	Intensity of Staining	Tracer Uptake
1	PA	n/a *	n/a	Strong
2	PA	n/a	n/a	Moderate
3	PA	55	3	Weak
4	PA	80	3	Moderate
5	PA	20	3	Moderate
6	Warthin tumor	0	0	Weak
7	Warthin tumor	0	0	None
8	Warthin tumor	0	0	None
9	Oncocytoma	0	0	Weak
10	B-NHL	20	2	Weak
11	SCC	0	0	None
12	Granuloma	n/a	n/a	None
13	Chronic parotitis	0	0	None

* n/a: Not available.

**Table 3:** Comparison of SUVmax and SUVmean.

	SUVmax			SUVmean		
	Tumor	Contralateral	*p*	Tumor	Contralateral	*p*
Pleomorphic adenoma	9.65 ± 6.34	1.53 ± 0.33	0.02	4.29 ± 2.38	1.15 ± 0.20	0.02
Warthin tumor	1.98 ± 0.63	1.81 ± 0.85	0.79	1.33 ± 0.68	1.33 ± 0.75	1.00
Other	3.67 ± 1.84	2.17 ± 0.44	0.11	2.61 ± 1.08	1.62 ± 0.25	0.08

**Table 4:** Comparison of liver and blood pool quotients.

	Liver Quotient			Blood Pool Quotient		
	Tumor	Contralateral	*p*	Tumor	Contralateral	*p*
Pleomorphic adenoma	1.20 ± 0.85	0.18 ± 0.04	0.03	11.40 ± 8.28	1.73 ± 0.60	0.03
Warthin tumor	0.24 ± 0.02	0.22 ± 0.05	0.50	3.05 ± 1.08	2.77 ± 1.36	0.80
Other	0.46 ± 0.25	0.27 ± 0.08	0.15	4.92 ± 2.94	2.91 ± 1.04	0.19

**Table 5:** Ratio comparison.

	Ratio-SUVmax	Ratio-SUVmean	Ratio-Liver Quotient	Ratio-Blood Pool Quotient
Pleomorphic adenoma	6.26 ± 4.31	3.60 ± 1.93	1.20 ± 0.85	11.40 ± 8.28
Warthin tumor	1.15 ± 0.16	1.03 ± 0.20	0.24 ± 0.02	3.05 ± 1.08
Other	1.81 ± 1.13	1.67 ± 0.85	0.46 ± 0.25	4.92 ± 2.94

## Data Availability

The original contributions presented in the study are included in the article, further inquiries can be directed to the corresponding author.
